# A murine cellular model of necroinflammation displays RAGE‐dependent cytokine induction that connects to hepatoma cell injury

**DOI:** 10.1111/jcmm.15649

**Published:** 2020-07-22

**Authors:** Malte Bachmann, Laura Lamprecht, Sina Gonther, Josef Pfeilschifter, Heiko Mühl

**Affiliations:** ^1^ pharmazentrum frankfurt/ZAFES Universitätsklinikum Frankfurt Goethe‐University Frankfurt am Main Frankfurt am Main Germany

**Keywords:** cytokines, hepatocellular carcinoma, high‐mobility group box‐1 (HMGB1), inflammation, necrosis, receptor for advanced glycation end product (RAGE)

## Abstract

Unresolved inflammation maintained by release of danger‐associated molecular patterns, particularly high‐mobility group box‐1 (HMGB1), is crucial for hepatocellular carcinoma (HCC) pathogenesis. To further characterize interactions between leucocytes and necrotic cancerous tissue, a cellular model of necroinflammation was studied in which murine Raw 264.7 macrophages or primary splenocytes were exposed to necrotic lysates (N‐lys) of murine hepatoma cells or primary hepatocytes. In comparison to those derived from primary hepatocytes, N‐lys from hepatoma cells were highly active—inducing in macrophages efficient expression of inflammatory cytokines like C‐X‐C motif ligand‐2 , tumor necrosis factor‐α, interleukin (IL)‐6 and IL‐23‐p19. This activity associated with higher levels of HMGB1 in hepatoma cells and was curbed by pharmacological blockage of the receptor for advanced glycation end product (RAGE)/HMGB1 axis or the mitogen‐activated protein kinases ERK1/2 pathway. Analysis of murine splenocytes furthermore demonstrated that N‐lys did not comprise of functionally relevant amounts of TLR4 agonists. Finally, N‐lys derived from hepatoma cells supported inflammatory splenic Th17 and Th1 polarization as detected by IL‐17, IL‐22 or interferon‐γ production. Altogether, a straightforward applicable model was established which allows for biochemical characterization of immunoregulation by HCC necrosis in cell culture. Data presented indicate a remarkably inflammatory capacity of necrotic hepatoma cells that, at least partly, depends on the RAGE/HMGB1 axis and may shape immunological properties of the HCC microenvironment.

## 1 INTRODUCTION

Necrosis and its related cell death entities necroptosis and pyroptosis initiate and/or perpetuate inflammation^1^, ^2^ thereby shaping the course of various diseases, among others hepatitides of diverse causes.^3^, ^4^ Specifically, hepatic ischaemia reperfusion injury^5^ and acetaminophen‐induced liver injury^5^, ^6^, ^7^ as well as alcoholic (ASH) and non‐alcoholic steatohepatitis (NASH)^4^, ^8^ are well‐described examples of acute or chronic hepatic diseases in which necroinflammation is at the epicentre of pathophysiology. By fuelling chronic inflammation, necrotic cell death is moreover key to progression of NASH/ASH or viral hepatitides to hepatocellular carcinoma (HCC).^8^, ^9^, ^10^

On a biochemical level, sterile inflammation induced by necrosis is mediated by release/misplacement of intracellular molecules normally invisible to receptors of innate immunity. According to the danger hypotheses, these danger‐associated molecular patterns (DAMPs) initiate inflammatory signalling by activating key innate receptor systems.^4^ Among DAMPs well characterized with regard to the inflamed liver are histones mainly targeting toll‐like receptors (TLR)‐2/4/9 (in case of TLR9 likely in combination with DNA),^11^, ^12^, ^13^ RNA targeting discrete endosomal nucleic acids‐sensing TLRs such as TLR3,^14^ and high‐mobility group box‐1 (HMGB1) targeting mainly the receptor for advanced glycation end products (RAGE).^15^, ^16^ Resultant cellular responses not only maintain inflammatory hepatic diseases but also drive hepatocellular carcinogenesis^8^, ^9^ for which accumulating evidence indicates pivotal action of RAGE signalling. Specifically, HMGB1/RAGE signalling has been implicated in sustaining an inflammatory tumour microenvironment that supports hepatic oval cell and carcinoma proliferation as well as tumour invasion and metastasis.^17^, ^18^, ^19^, ^20^

Herein, we established a feasible murine cell culture model that allows for investigation of immunoregulation by necrotic HCC cells under defined conditions. Data presented prove a highly inflammatory nature of necrotic HCC cells which translates into (at least) partly RAGE/HMGB1‐dependent up‐regulation of cytokine production. Among those are C‐X‐C motif ligand‐2 (CXCL2), tumour necrosis factor (TNF)‐α, interleukin (IL)‐36α, IL‐23‐p19 and IL‐17 which jointly have the capability to maintain an inflammatory pro‐cancerous micromilieu in the diseased liver tissue.

## 2 MATERIALS AND METHODS

### 2.1 Reagents

Murine agonistic αCD3 (#17A2) antibody was purchased from Biolegend (San Diego, CA, USA). Antagonistic recombinant box‐A domain (box‐A) from HMGB1 (HM‐012) was from TECAN (Männedorf, Switzerland). The RAGE antagonist FPS‐ZM1 (herein denoted Ri), the mitogen‐activated protein kinase (MAPK) kinase (MEK) inhibitor U0126, the HMGB1 antagonist glycyrrhizin (Gly) and phorbol 12‐myristate 13 acetate (PMA) were from Merck/Millipore (Darmstadt, Germany). Human IL‐1β, murine IL‐2 and murine IL‐6 were obtained from Peprotech Inc (Frankfurt, Germany). Anti‐murine IL‐4 antibody, murine IL‐12, IL‐23 and TGFβ were from R&D Systems (Wiesbaden, Germany). Endotoxin (LPS) (O55:B5, TLR grade) was purchased from Sigma‐Aldrich (Taufkirchen, Germany). TLR9 agonistic type‐B ODN1826 and TLR9 antagonist ODN2088 were from Invivogen (San Diego, CA, USA).

### 2.2 Cultivation of hepatoma cells (Hepa1‐6, Hep‐56.1D, HepG2), RAW 264.7 macrophages and THP1 cells

Hepa1‐6 (LGC Standards, Wesel, Germany) and Hep‐56.1D (CLS GmbH, Eppenheim, Germany) murine hepatoma cell lines, and human hepatoma HepG2 cells (LGC Standards) were maintained in DMEM supplemented with 100 U/mL penicillin, 100 μg/mL streptomycin and 10% heat‐inactivated foetal calf serum (FCS) (Thermo Fisher Scientific, Langenselbold, Germany). For experiments, cells were seeded on six‐well polystyrene plates (Greiner, Frickenhausen, Germany).

RAW 264.7 macrophages (CLS GmbH) and monocytic THP1 cells (German Collection of Microorganisms and Cell Cultures, Braunschweig, Germany) were maintained in roswell park memorial institute (RPMI) 1640 supplemented with 100 U/mL penicillin, 100 μg/mL streptomycin and 10% heat‐inactivated FCS. For experiments, cells were seeded on six‐well polystyrene plates. For RAW 264.7 macrophages, 2.5 x 10^5^ cells/1 mL were seeded per well. Experiments were started (after medium change) 34 or 48 hours thereafter (for experiments using an incubation period of 16 or 6 hours). THP1 cells were seeded at 10^6^ cells/2 mL. To differentiate THP1 cells into macrophage‐like adherent cells those were incubated for 16 hours in aforementioned medium with PMA (50 ng/mL). Thereafter, cells were washed with PBS and kept in medium for recovery. After 24 hours, cells were washed twice with PBS followed by stimulation in medium under conditions indicated. Here, ‘ctrl’ denotes PMA‐differentiated THP1 cells (mTHP1) without any further stimulation.

### 2.3 Isolation and cultivation of primary murine hepatocytes

Male C57Bl/6J mice (MFD Diagnostics, Wendelsheim, Germany; 9‐12 weeks old) were sacrificed, and obtained livers were perfused post‐mortem. Briefly, perfusion was performed with 42°C warm HBSS—without Ca^2+^ and Mg^2+^—(supplemented with 15 mmol/L 4‐(2‐hydroxyethyl)‐1‐piperazineethanesulfonic acid (HEPES) , 2.5  mmol/L ethylene glycol‐bis(2‐aminoethylether)‐N,N,N',N'‐tetraacetic acid (EGTA), 1 g/L glucose, 1× non‐essential amino acids (Sigma‐Aldrich, Darmstadt, Germany), 100 U/mL penicillin, and 100 µg/mL streptomycin) using a roller pump (10 mL/min) for 10 minutes. Thereafter, livers were perfused with HBSS with Ca^2+^ and Mg^2+^ (supplemented with 15 mmol/L HEPES, 5 mmol/L CaCl_2_, 1× non‐essential amino acids [Sigma‐Aldrich], 0.13 mg/mL collagenase IV [Sigma‐Aldrich]) for additional 10 minutes. Livers were carefully removed from the abdominal cavity, placed in Petri dishes on ice in DMEM (supplemented with 10% FCS, 100 U/mL penicillin and 100 µg/mL streptomycin) and opened with forceps. Liver cells were resuspended and put over a 100 µm cell strainer (Becton Dickinson, Heidelberg, Germany). After two rounds of centrifugation (5 minutes at 50 *g* and 4°C) and resuspension, cell viability was determined by trypan blue dye exclusion and cells were seeded in DMEM (supplemented with 10% FCS, 100 U/mL penicillin, and 100 µg/mL streptomycin) on collagen G‐coated plates (Biochrom, Berlin, Germany). Adherent hepatocytes were washed after 4 hours with PBS and fresh Williams’ Medium E (supplemented with 10% heat‐inactivated FCS, 2 mmol/L L‐alanyl‐L‐glutamine (Biochrom), 2 ng/mL insulin, 100 U/mL penicillin and 100 µg/mL streptomycin) was added. Cultivation was performed at 37°C and 5% CO_2_. Preparation of lysates was performed 16 hours thereafter.

### 2.4 Isolation of primary murine splenocytes

Spleens were obtained from 9‐ to 12‐week‐old wild‐type male C57Bl/6J mice (MFD Diagnostics, Wendelsheim, Germany). Where indicated, TLR4‐deficient mice and their respective wild‐type controls (both C57Bl/6J, Zentrale Forschungseinrichtung, Universitätsklinikum Frankfurt; kindly provided by Prof. Liliana Schäfer, *pharmazentrum frankfurt*) were used to isolate splenocytes. Spleens were excised and transferred to 5 mL ice‐cold RPMI 1640 medium. Tissue was destroyed over a nylon cell strainer (70 μm; BD Biosciences, Heidelberg, Germany). Cell suspensions were centrifuged at 500 *g* for 5 minutes at 4°C and resuspended in 2 mL 0.83% NH_4_Cl for 2 minutes at room temperature. Red blood cell lysis was stopped by adding 10 mL cold RPMI 1640 medium. Splenocytes were collected by centrifugation, washed once with RPMI and resuspended in RPMI 1640 supplemented with 10% heat‐inactivated FCS and 100 U/mL penicillin, 100 μg/mL streptomycin. 3 × 10^6^ cells were seeded on 24‐well polystyrene plates (Greiner) in 0.5 mL culture medium.

### 2.5 Th1 and Th17 differentiation in splenocytes

To induce polarization in the splenocyte T cell population, splenocytes were resuspended in RPMI 1640 (supplemented with 10% heat‐inactivated FCS and 100 U/mL penicillin, 100 µg/mL streptomycin) and seeded onto six‐well plates coated with anti‐murine CD3 antibody (4 µg/mL). For Th1 differentiation cells were maintained in presence of murine IL‐2 (20 ng/mL), IL‐12 (20 ng/mL) and αIL‐4 antibodies (5 µg/mL). For Th17, differentiation splenocytes were maintained in presence of murine IL‐6 (20 ng/mL), IL‐23 (6 ng/mL) and TGFβ (3 ng/mL). After 4 days, supernatants were collected and assayed for production of indicated cytokines.

### 2.6 Preparation of necrotic cell lysates from murine/human hepatoma cells and primary murine hepatocytes

Confluent adherent cell cultures (Hepa1‐6 cells, Hep‐56.1D, HepG2, primary murine hepatocytes) were washed twice with PBS followed by three cycles of freeze/thawing (at −80°C/+37°C) in RPMI supplemented with 100 U/mL penicillin and 100 µg/mL streptomycin. Thereafter, remaining adherent cells on polystyrene plates were scraped off followed by centrifugation at 17000 g (4°C, 10 minutes). Insoluble pellets were discarded, whereas supernatants were aliquoted and denoted necrotic lysates (N‐lys) when derived from Hepa1‐6 cells or Pri‐N‐lys when derived from primary hepatocytes or N‐lys‐HepG2 when derived from HepG2 cells. For storage (at −80°C), 10% FCS was added to the lysates.

DNA content is regarded a most accurate correlate reflecting epithelial cell numbers. Accordingly, a PicoGreen‐based highly sensitive and reliable method was applied for quantification of the dsDNA content in aforementioned cellular lysates. For that purpose, the Quant‐iT PicoGreen assay kit was used according to the manufacturer's instructions (Thermo Fisher Scientific). Obtained dsDNA concentrations were used for sample calibration (in ng/mL) which enabled usage of cell lysates as stimulus for cultured cells.

### 2.7 Cytokine release detected by ELISA

Concentration of murine IFNγ, IL‐6, IL‐17A (herein denoted IL‐17), IL‐22, CXCL2 (MIP2) (all DuoSet ELISA, R&D Systems), murine TNFα (eBioscience, Frankfurt, Germany) and human IL‐8 (CXCL8) (BDBiosciences, Heidelberg, Germany) in cell‐free culture supernatants was determined by ELISA. Assays were performed according to the manufacturers' instructions.

### 2.8 Immunoblot analysis

For detection of phosphorylated (activated) and total ERK1/ERK2 (p42/p44), whole‐cell lysates were generated using lysis buffer (150 mmol/L NaCl, 1 mmol/L CaCl2, 25 mmol/L Tris‐Cl [pH 7.4], 1% Triton X‐100) supplemented with protease inhibitor cocktail (Roche Diagnostics) and DTT/Na_3_VO_4_/PMSF (each 1 mmol/L) and with NaF (20 mmol/L). Thereafter, SDS‐PAGE and immunoblotting were performed using 50 μg of total protein applied per lane. For detection of total p44/42, blots were stripped and reprobed. Antibodies used: p‐p44/p‐p42 (rabbit polyclonal, #9101; Cell Signalling, Frankfurt, Germany), total p44/p42 (rabbit polyclonal, #9102, Cell Signaling). For detection of HMGB1, cell lysate from Hepa1‐6 and RAW 264.7 cells as well as N‐lys from Hepa1‐6 cells (N‐lys), Hep‐56.1D cells, and primary hepatocytes (Pri‐N‐lys) were subjected to SDS‐PAGE and immunoblotting. The amount of necrotic lysate that was applied per lane equalled 20 ng of (necrotic) dsDNA. Antibody: HMGB1 (rabbit polyclonal, #10829‐1‐AP; Proteintech, Rosemont, USA).

### 2.9 Quantification of mRNA expression by Real‐time PCR

Total RNA, isolated by TRI Reagent (Sigma‐Aldrich) was transcribed using random hexameric primers (Qiagen, Hilden, Germany) and Moloney virus reverse transcriptase (Life Technologies, Darmstadt, Germany) according to the manufacturers' instructions. During real‐time PCR, changes in fluorescence were caused by the Taq polymerase degrading the probe that contains a fluorescent dye (glycerinaldehyde‐3‐phosphate‐dehydrogenase [GAPDH]: VIC, all others: FAM; Life Technologies). Pre‐developed reagents: GAPDH (4352339E), IL‐6 (Mm00446190_m1), IL‐10 (Mm01288386_m1), IL‐36α (Mm00457645_m1), IL‐36γ (Mm00463327_m1), IL‐23p19 (Mm00518984_m1), Irg1 (Mm01224532_m1), CXCL2 (Mm00436450_m1), TNFα (Mm00443258_m1). Assay mix was from Nippon Genetics (Düren, Germany). Real‐time PCR was performed (according to the manufacturers' instructions on a AbiPrism7500 Fast Sequence Detector (Life Technologies): One initial steps at 95°C (2 minutes) was followed by 40 cycles at 95°C (5 seconds) and 62°C (30 seconds). Detection of the dequenched probe, calculation of threshold cycles (Ct values) and data analysis were performed by the Sequence Detector software. Relative changes in mRNA expression compared to unstimulated control and normalized to GAPDH were quantified by the 2^−ddCt^ method.

### 2.10 Quantification of viable cells by WST‐1 assay

RAW 264.7 macrophages or splenocytes were seeded onto 96‐well polystyrene plates. Cells were stimulated as indicated or cultured as unstimulated control. After the indicated time‐points, viable cells were quantified using WST‐1 assay reagent (Roche Diagnostics) according to the manufacturer's instructions.

### 2.11 Statistical analysis

Data are shown as means ± SD or as means ± SEM (as indicated) and presented as fold induction, pg/mL, ng/mL, or as per cent of N‐lys alone. Statistical analysis was performed as indicated in the legends by one‐way ANOVA with post hoc Bonferroni correction (GraphPad 5.0) or unpaired Student's *t* test.

## 3 RESULTS

### 3.1 Immunoactivation and cytokine responses detected in RAW 264.7 macrophages under the influence of necrotic hepatoma lysates

**FIGURE 1:**
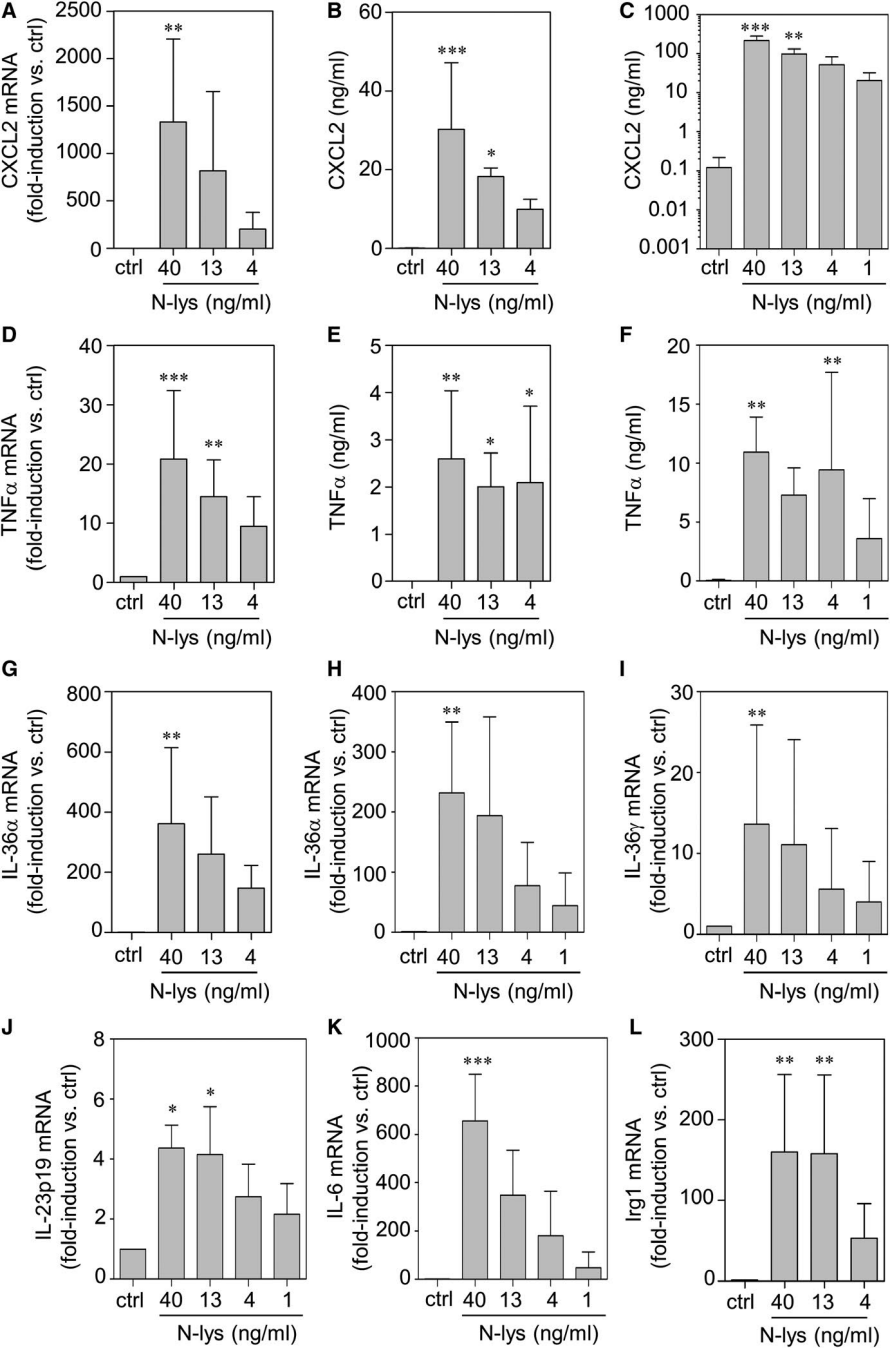
RAW 264.7 macrophages were kept as unstimulated control or stimulated with the indicated concentration of N‐lys for 6 h (A, B, D, E, G, L) or 16 h (C, F, H‐K). mRNA expression of indicated genes (A, D, G‐L) was determined by real‐time PCR, normalized to that of glycerinaldehyde‐3‐phosphate‐dehydrogenase and is shown as mean fold induction compared to unstimulated control ± SD (A, D, G‐K: n = 4‐5; L: n = 3). *P < 0.05, **P < 0.01, ***P < 0.001 compared to unstimulated control; raw data were analysed by one‐way ANOVA with post hoc Bonferroni correction. Release of CXCL2 (B, C) or TNFα (E, F) was determined by ELISA. Data are shown as means ± SD (B, C: n = 5; E, F: n = 4‐7). *P < 0.05, **P < 0.01, ***P < 0.001 compared to unstimulated control; statistical analysis, one‐way ANOVA with post hoc Bonferroni correction

In order to characterize immunoregulatory/stimulatory properties of cellular material derived from necrotic hepatoma tissue, murine RAW 264.7 macrophages were exposed to increasing amounts of necrotic cell lysates derived from murine hepatoma Hepa1‐6 cells (N‐lys). Exposure at the indicated concentrations did not significantly affect cell viability (100.6 ± 24.1% compared to unstimulated cells set as 100%; N‐lys (40 ng/mL, 16 hours, n = 3). As shown in Figure 1, N‐lys was highly potent in inducing mRNA expression (A) and release (BC) of the prototypic inflammatory chemokine CXCL2 (MIP‐2)^21^ in dose‐ and time‐dependent manner. Moreover, cytokine expression of inflammatory TNFα (DEF), IL‐36α/γ (GHI) as well as IL‐23‐p19 (J) and IL‐6 (K) was significantly up‐regulated by N‐lys. In accord with N‐lys being a potent macrophage activator, up‐regulation of modulatory Irg1^22^ was likewise detectable in these same cultures (L). Stimulatory properties of N‐lys were not restricted to Hepa1‐6 cells as hepatoma source. Necrotic cell lysates derived from the alternative murine hepatoma cell line Hep‐56.1D actually displayed very similar inflammatory properties (CXCL2 secretion [24 hours]: 0.2 ± 0.2 ng/mL vs 246.1 ± 59.4 ng/mL for unstimulated control vs stimulation with N‐lys [equalling 40 ng/mL DNA], n = 3, *P* < .01).

Immunostimulatory characteristics of necrotic hepatoma tissue were not confined to the murine system. Specifically, necrotic cell lysates derived from human hepatoma HepG2 cells were likewise able to potently activate human macrophage‐like THP1 (mTHP1) cells as detected by analysis of IL‐8 release (Figure S1).

### 3.2 Macrophage activation by necrotic hepatoma lysates is dependent on RAGE and ERK1/2 signalling

**FIGURE 2:**
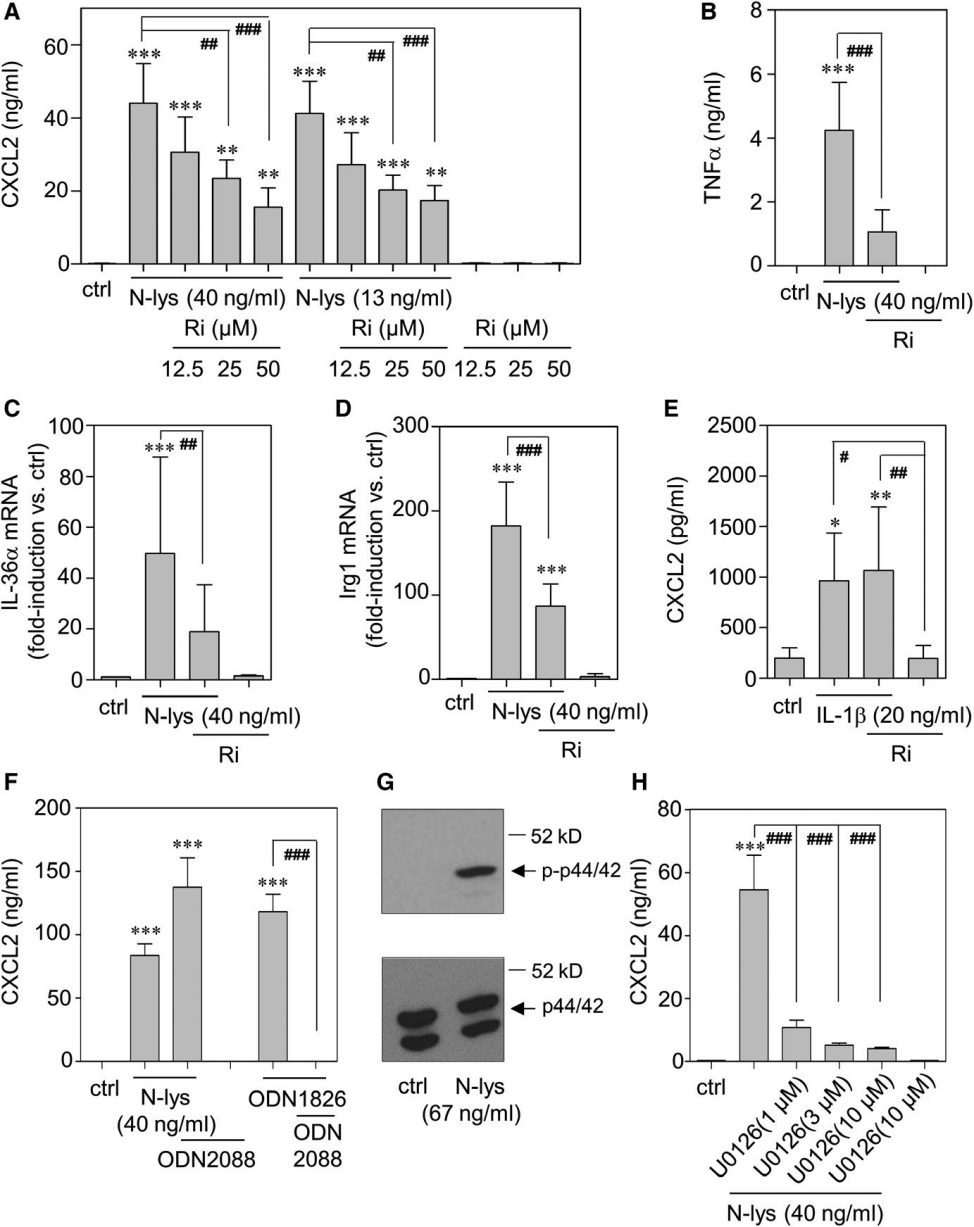
A‐E, H: RAW 264.7 macrophages were kept as unstimulated control or stimulated with N‐lys (as indicated 40 or 13 ng/mL) or IL‐1β (20 ng/mL) for 6 h. Where indicated, cells were pre‐incubated with Ri or U0126 (at indicated concentrations) for 30 min. All cultures were adjusted to a final concentration of 0.03% dimethyl sulfoxide (DMSO) (vehicle for Ri) or 0.1% DMSO (vehicle for U0126). Secreted CXCL2 (A, E, H) or TNFα (B) was determined by ELISA. Data are shown as means ± SD (A: n = 3‐9, B: n = 9, E: n = 6, H: n = 3). *P < 0.05, **P < 0.01, ***P < 0.001 compared to unstimulated control; ^#^ *P* < 0.05, ^##^ *P* < 0.01, ^###^ *P* < 0.001; statistical analysis, one‐way ANOVA with post hoc Bonferroni correction. IL‐36α (C) and Irg1 (D) mRNA was determined by real‐time PCR, normalized to that of GAPDH, and is shown as mean fold induction compared to unstimulated control ± SD (C: n = 7‐8, D: n = 4‐5). ***P < 0.001 compared to unstimulated control, ^##^ *P* < 0.01, ^###^ *P* < 0.001; raw data were analysed by one‐way ANOVA with post hoc Bonferroni correction. F, RAW 264.7 macrophages were kept as unstimulated control or stimulated N‐lys (40 ng/mL) or with agonistic B‐type ODN1826 (2 µmol/L) for 16 h. Where indicated, cells were pre‐incubated with antagonist ODN2088 (10 µmol/L) for 30 min. Secreted CXCL2 was determined by ELISA. Data are shown as means ± SD (n = 3). ***P < 0.001 compared to unstimulated control; ^###^ *P* < 0.001; statistical analysis, one‐way ANOVA with post hoc Bonferroni correction. G, RAW 264.7 macrophages were kept as unstimulated control or were stimulated with 67 ng/mL N‐lys. After 10 min, cellular content of p‐p44/p‐p42 and total p44/p42 was determined by immunoblot analysis. One representative of three independently performed experiments is shown

As already referred to, RAGE signalling is supposed to substantially impact inflammatory liver carcinogenesis.^16^, ^23^ In order to determine the relevance of RAGE concerning macrophage activation by N‐lys, RAW 264.7 cells were exposed to RAGE antagonistic FPS‐ZM1 (herein denoted Ri). As shown in Figure 2, release of CXCL2 was dose‐dependently inhibited upon pre‐incubation (30 minutes) with Ri (A). Exposure of RAW 264.7 to Ri at the indicated concentrations did not affect cell viability (100.9 ± 22.8% for cells stimulated with N‐lys [40 ng/mL]/Ri [50 μmol/L, 30 minutes pre‐incubation] compared to those stimulated with N‐lys alone [40 ng/mL] set as 100% [6 hours incubation, n = 3]). Expression of further N‐lys‐inducible genes was likewise impaired by RAGE antagonism which is shown for TNFα (B), IL‐36α (C) and IRG1 (D). In contrast, IL‐1β‐induced CXCL2 remained unaffected by Ri (E). Since TLR9‐activating cellular DNA may serve as biologically active DAMP^11^ in this experimental setting, outcome of macrophage pre‐incubation (30 minutes) with the TLR9 antagonist ODN2088 was evaluated. Whereas TLR9‐activating B‐type CpG‐ODN1826‐induced CXCL2 was nullified in presence of ODN2088, this compound failed to significantly affect release of this chemokine under the influence N‐lys (Figure 2F).

Because potent activation of MAPK is a key feature of RAGE signal transduction,^24^ the relevance of this pathway was investigated by its blockage using the MEK inhibitor U0126. In fact, activation by N‐Lys strongly stimulated ERK1/2 activation (Figure 2G) and pre‐incubation (30 minutes) with U0126 actually suppressed associated CXCL2 secretion (Figure 2H).

### 3.3 HMGB1 serves inflammatory functions in necrotic hepatoma lysates

**FIGURE 3:**
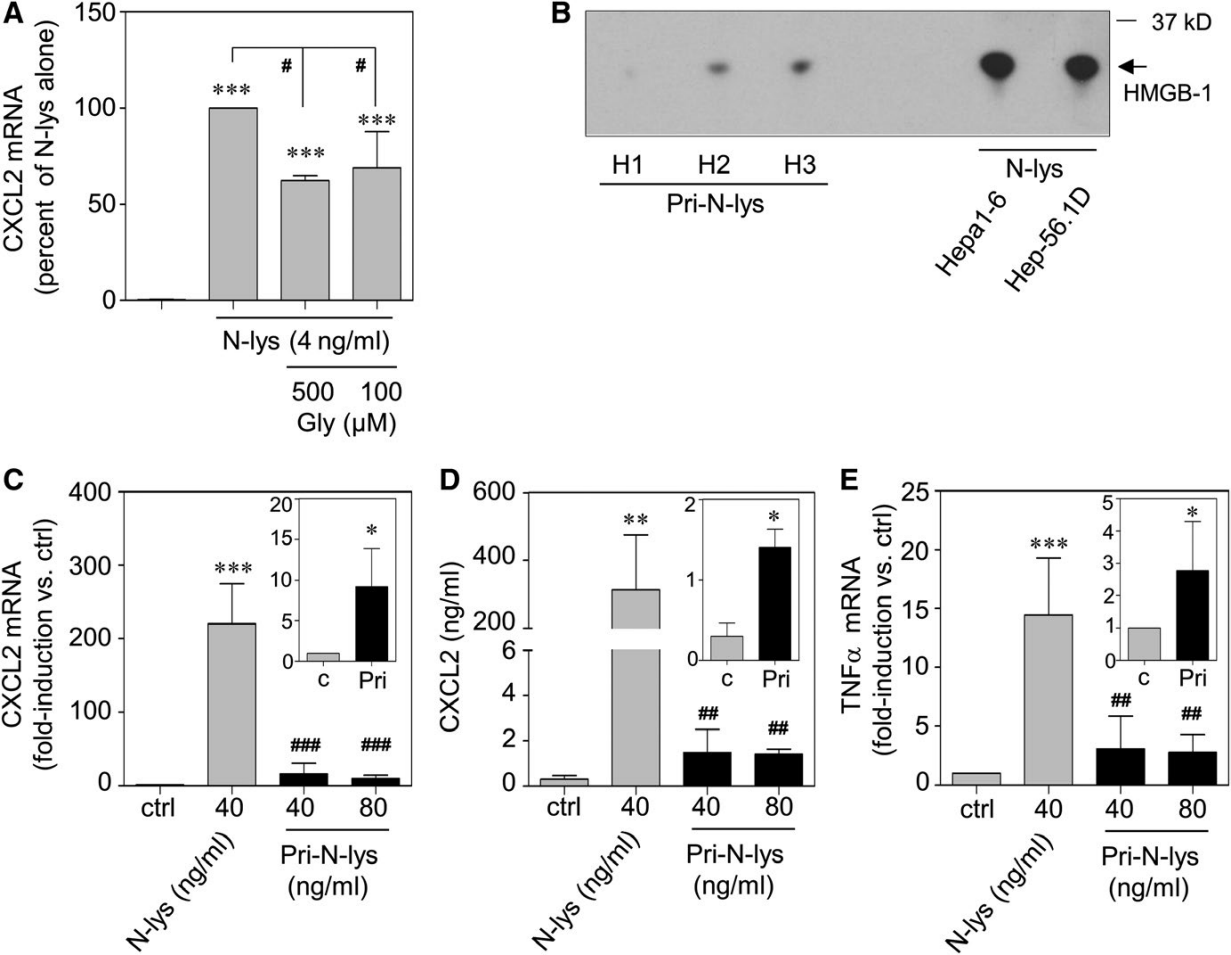
A, RAW 264.7 macrophages were kept as unstimulated control or were stimulated with 4 ng/mL N‐lys for 16 h. Where indicated, N‐lys was pre‐treated with glycyrrhizin (Gly; 500 or 100 µmol/L, final concentrations on cells) for 30 min. All cultures were adjusted to a final concentration of 0.4% DMSO (vehicle for Gly). Secreted CXCL2 was determined by ELISA. Data are shown as means ± SD (n = 5). ***P < 0.001 compared to unstimulated control; ^#^ *P* < 0.05; statistical analysis, one‐way ANOVA with post hoc Bonferroni correction. B, Content of HMGB‐1 in N‐Lys or Pri‐N‐lys (equalling 20 ng of DNA) was determined by immunoblot analysis. C‐E, RAW 264.7 macrophages were kept as unstimulated control or stimulated with 40 ng/mL N‐lys or indicated concentrations of Pri‐N‐lys (40 or 80 ng/mL) for 24 h. CXCL2 (C) and TNFα (E) mRNA was determined by real‐time PCR, normalized to that of GAPDH, and is shown as mean fold induction compared to unstimulated control ± SD (n = 3; ***P < 0.001 compared to unstimulated control; ^##^ *P* < 0.01, ^###^ *P* < 0.001 compared to N‐lys; raw data were analysed by one‐way ANOVA with post hoc Bonferroni correction. Insets: mRNA expression of CXCL2 (C) or TNFα (E) by Pri‐N‐lys (Pri, 80 ng/mL) stimulated or unstimulated RAW 264.7 macrophages is shown at a larger *y*‐axis scale. *P < 0.05 compared to unstimulated control; raw data were analysed by unpaired *t* test. D: Secreted CXCL2 was determined by ELISA. Data are shown as means ± SD (n = 3). **P < 0.01 compared to unstimulated control; ^##^ *P* < 0.01 compared to N‐lys; raw data were analysed by one‐way ANOVA with post hoc Bonferroni correction. Inset: Secreted CXCL2 by Pri‐N‐lys (Pri, 80 ng/mL) stimulated or unstimulated RAW 264.7 macrophages is shown at a larger *y*‐axis scale. *P < 0.05 compared to unstimulated control; raw data were analysed by unpaired Student's *t* test

HMGB1 is a key DAMP and agonistic RAGE ligand that potently initiates RAGE‐dependent inflammatory signalling.^16^, ^23^ In order to determine the functional relevance of HMGB1 in the context of pro‐inflammatory N‐lys properties, lysates were pre‐incubated with RAGE antagonist glycyrrhizin (Gly).^25^ Viability of RAW 264.7 cells was not affected by Gly at chosen concentrations.^26^ In fact, pharmacological inhibition of HMGB1 by this compound significantly reduced N‐Lys‐mediated CXCL2 release by 37.6% at 500 μmol/L (Figure 3A). Recombinant box‐A domain of HMGB1 was used as a further specific HMGB1 antagonist.^15^ Notably, pre‐incubation with recombinant box‐A domain resulted in inhibitory effects very similar to those of Gly (Figure S2). Accordingly, HMGB1 was readily detectable by immunoblot analysis in N‐lys obtained from Hepa1‐6 or Hep5.1D cells. Immunoblot analysis likewise revealed that, compared to N‐lys derived from aforementioned hepatoma cells, those derived from primary murine hepatocytes (Pri‐N‐lys) displayed considerably less HMGB1 protein expression (Figure 3B). Specifically, hepatocyte isolates from three individual C57Bl/6J mice were analysed (H1‐H3). Of note, longer exposure times revealed well‐detectable HMGB1 expression also in the lysate denoted H1 (data not shown). Those observations precisely confirm a previous report demonstrating that Hepa1‐6 cells in fact express far higher levels of HMGB1 protein as compared to primary hepatocytes.^17^ Remarkably, Hepa1‐6 hepatoma cells were also found to express far higher levels of HMGB1 as compared to RAW264.7 macrophages (Figure S3).

In order to test whether different levels of HMGB1 in cellular lysates may affect their immunostimulatory properties, RAW 264.7 macrophages were exposed to N‐lys (from Hepa1‐6 cells) or to each of the lysates derived from aforementioned primary hepatocytes (Pri‐N‐lys H1 to H3 reflecting n = 3 in Figure 3C‐E). In concurrence with the view of HMGB1 contributing to inflammatory functions of N‐lys, experiments revealed that, as compared to Pri‐N‐lys, N‐lys was considerably more potent in stimulating expression of CXCL2 (Figure 3C,D) or TNFα (Figure 3E). Notably, albeit on a much lower level, Pri‐N‐lys induced significant inflammatory CXCL2 and TNFα production (Figure 3, see insets).

### 3.4 Immunostimulatory properties of necrotic hepatoma lysates as detected in murine splenocytes

**FIGURE 4:**
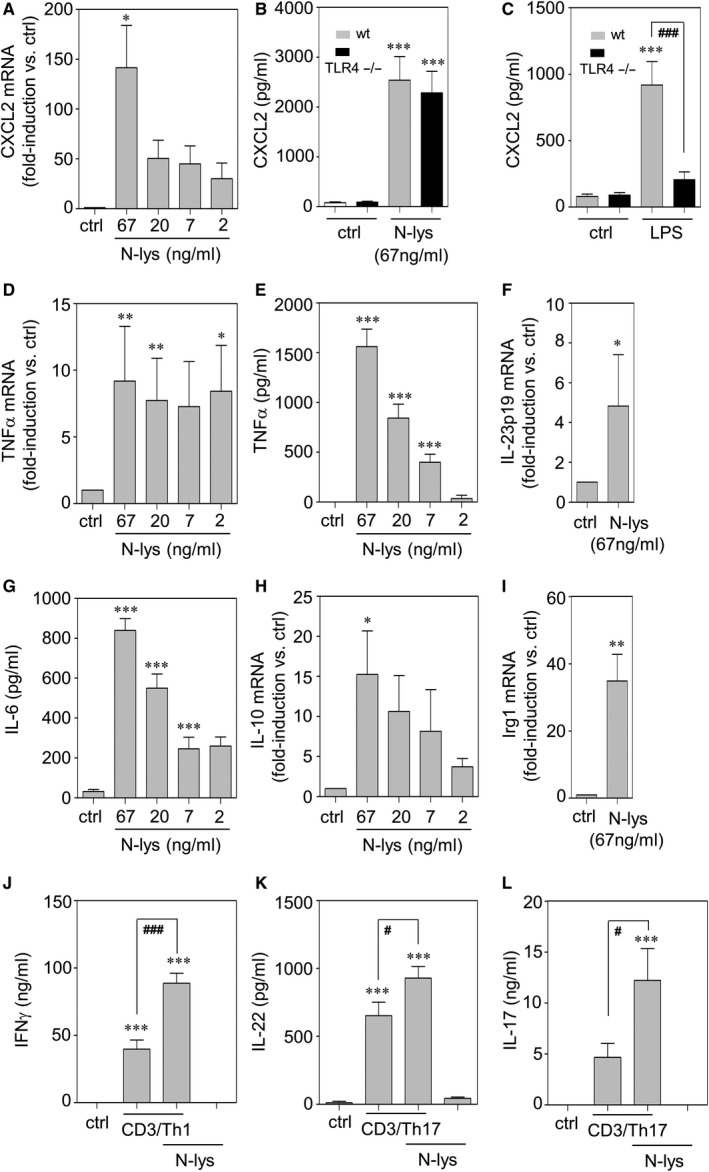
Splenocytes of male C57BL/6J wt mice were kept as unstimulated control or were stimulated with indicated concentrations of N‐lys for 6 h (A, F) or 16 h (D, E, G, H, I). mRNA (A, D, F, H, I) for indicated genes was determined by real‐time PCR, normalized to that of GAPDH, and is shown as mean fold induction compared to unstimulated control ± SEM (A: n = 4‐5; D: n = 5; F: n = 6; H: n = 5; I: n = 7). *P < 0.05, **P < 0.01 compared to unstimulated control; raw data were analysed by one‐way ANOVA with post hoc Bonferroni correction (A, D, H) or unpaired Student's *t* test (F, I). Secreted TNFα (E) or IL‐6 (G) was determined by ELISA. Data are shown as means ± SEM (E: n = 5; G: n = 3‐5). ***P < 0.001 compared to unstimulated control; statistical analysis, one‐way ANOVA with post hoc Bonferroni correction. B and C, Splenocytes of male C57BL/6J wt mice or male TLR4 deficient mice were kept as unstimulated control or were stimulated with N‐lys (B, 67 ng/mL) or LPS (C, 1 µg/mL) for 16 h. Secreted CXCL2 was determined by ELISA. Data are shown as means ± SEM (n = 3). ***P < 0.001 compared to unstimulated control of the same genotype, ^###^ *P* < 0.001; statistical analysis, one‐way ANOVA with post hoc Bonferroni correction. J‐L, Splenocytes of male C57BL/6J wt mice were kept as unstimulated control or were cultivated under Th1 (J) or Th17 conditions (K, L) with or without N‐lys (67 ng/mL). After 4 d, secreted IFNγ (J), IL‐22 (K) and IL‐17 (L) were determined by ELISA. Data are shown as means ± SEM (J, L: n = 5, K: n = 6). ***P < 0.001 compared to unstimulated control, ^#^ *P* < 0.05, ^###^ *P* < 0.001; statistical analysis, one‐way ANOVA with post hoc Bonferroni correction

In order to investigate characteristics of N‐lys against the background of a naturally occurring murine leucocyte composition, the cellular model of freshly isolated splenocytes was applied. In fact, N‐lys efficiently stimulated induction of CXCL2 by splenocytes which was observed on mRNA and protein level (Figure 4A,B). By investigating splenocytes derived from TLR4‐deficient mice, a potential LPS contamination of N‐lys was excluded (Figure 4B). As expected, in these same experiments LPS‐induced CXCL2 release was nullified in TLR4^−/−^ mice (Figure 4C). A variety of further indicators of immunoactivation were up‐regulated by N‐lys in a manner similar to CXCL2. Those included TNFα (Figure 4D,E), IL‐23‐p19 (Figure 4F), IL‐6 (Figure 4G), IL‐10 (Figure 4H) and IRG1 (Figure 4I). Finally, inflammatory polarization of splenic T cells for Th1‐ and Th17‐like activation was enhanced under the influence of N‐lys, a phenomenon well detectable by amplified production of respective signature cytokines, namely IFNγ (Figure 4J), IL‐22 (Figure 4K) and IL‐17 (Figure 4L).

## 4 DISCUSSION

By using an in vitro cell culture model, we demonstrate an augmented inflammatory potential of murine necrotic hepatoma cells that, at least partly, relies on the RAGE/HMGB1 axis. Albeit in principle capable of mediating inflammatory macrophage activation, N‐lys derived from primary hepatocytes displayed far modest biological activity in this context. Those functional differences associated with strikingly divergent HMGB1 protein expression detectable in hepatoma cells and primary hepatocytes, respectively (Figure 3 and reference 17). Notably, HMGB1 which connects to inflammatory carcinogenesis^19^, ^20^, ^21^, ^22^ is increased in cancerous HCC tissues as well as in patients’ sera and serves as prognostic marker indicating progression of clinical HCC.^17^, ^27^, ^28^, ^29^, ^30^, ^31^, ^32^, ^33^ Notably, HMGB1 antagonizing glycyrrhizin and recombinant box‐A domain only partly abolished inflammatory action of N‐lys which concurs with the view that RAGE is a multi‐ligand receptor^23^ likely activated by further, to be identified, DAMPs present in N‐lys. In that context, it will be interesting in coming studies to characterize in detail differential properties of hepatic necroinflammation in cancerous vs non‐cancerous settings. This should include identification of crucial DAMPs and their potential to regulate associated immunoactivation.

A focus of the current study was to characterize in murine RAW 264.7 macrophages and freshly isolated splenocytes cytokine responses initiated by necroinflammation under the influence of N‐lys. In that context, we report on a remarkably potent gene induction of CXCL2 which is regarded a murine functional homologue of IL‐8 and was previously identified as crucial for neutrophil‐driven experimental HCC.^34^ Interestingly, CXCL2 likewise correlates with progression in HCC patients.^35^ We, herein, could also show that lysates from human hepatoma HepG2 cells likewise mediate potent IL‐8 release by human macrophage‐like mTHP1 cells. Furthermore, expression of TNF,^36^, ^37^, ^38^, ^39^ IL‐6,^40^, ^41^, ^42^, ^43^, ^44^, ^45^ IL‐10^46^, ^47^, ^48^ and IL‐23‐p19,^49^, ^50^ all of which connect to progression of experimental HCC and/or patients' prognosis, was up‐regulated by N‐lys‐stimulated murine RAW 264.7 macrophages and splenocytes. Interestingly, a recent study directly relates HMGB1 induction upon hypoxia to up‐regulation of macrophage IL‐6 and subsequent metastasis of murine HCC.^51^ IL‐36α/γ was inducible by N‐lys only in RAW 264.7 macrophages. Lack of IL‐36 regulation in splenocytes (data not shown) is surprising and a matter of further investigation. Finally, we demonstrate that N‐lys supports inflammatory T cell polarization as detected by enhanced expression of the Th1/Th17 signature cytokines IFNγ, IL‐22 and IL‐17. Although IFNγ displays complex immunoregulatory characteristics that include induction of potentially tumour‐supporting IL‐18 binding protein,^52^, ^53^ its tumour‐suppressive properties apparently override in the context HCC.^54^ In contrast, Th17‐derived IL‐17 which is supported by tumour‐associated macrophages clearly promotes progression in experimental and clinical HCC.^55^, ^56^, ^57^ Similarly, IL‐22 is a well‐defined pathogenic factor in HCC that likewise indicates patients' prognosis.^58^, ^59^, ^60^

Altogether, the cell culture model used herein validates a strong inflammatory potential of necrotic hepatoma cells that, at least partly, depends on the RAGE/HMGB1 axis and mediates production of key cytokines known to determine progression of HCC. Further biochemical analysis of necrotic normal hepatocytes vs necrotic cancerous hepatoma cells may sheet further light on the role that necroinflammation plays in HCC pathogenesis.

## CONFLICT OF INTEREST

The authors confirm that there are no conflicts of interest.

## AUTHOR CONTRIBUTION

**Malte Bachmann:** Data curation (equal); Formal analysis (equal); Investigation (lead); Methodology (equal); Project administration (supporting); Supervision (supporting); Writing‐original draft (equal); Writing‐review & editing (equal). **Laura Lamprecht:** Formal analysis (equal); Investigation (lead); Writing‐review & editing (equal). **Sina Gonther:** Investigation (supporting); Methodology (supporting); Writing‐review & editing (supporting). **Josef Pfeilschifter:** Formal analysis (supporting); Writing‐review & editing (equal). **Heiko Mühl:** Conceptualization (lead); Data curation (equal); Formal analysis (lead); Funding acquisition (lead); Methodology (equal); Project administration (lead); Supervision (lead); Writing‐original draft (lead); Writing‐review & editing (lead).

## Supporting information

Fig S1

Fig S2

Fig S3

## Data Availability

The data that support the findings of this study are available from the corresponding author upon reasonable request.
