# Supplementary figures and images for: A murine cellular model of necroinflammation displays RAGE‐dependent cytokine induction that connects to hepatoma cell injury
Source: J Cell Mol Med. 2020 Jul 22;24(18):10356–66. doi: 10.1111/jcmm.15649 (PMC7521286; doi:10.1111/jcmm.15649)

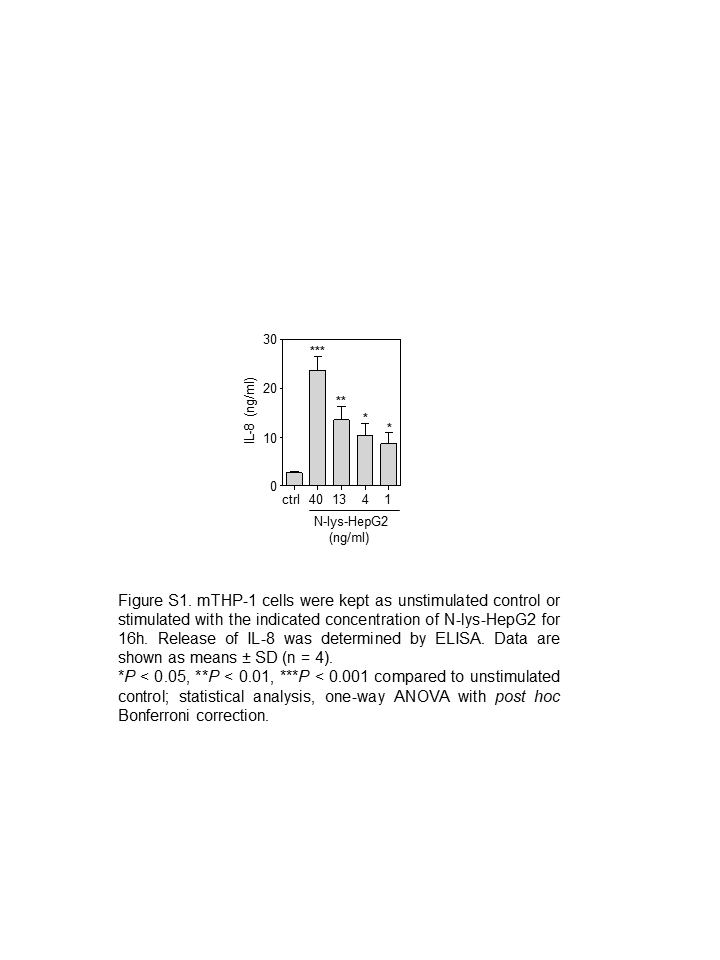

Supplement: Supplementary file 1 — Fig S1 [file JCMM-24-10356-s001.tif]

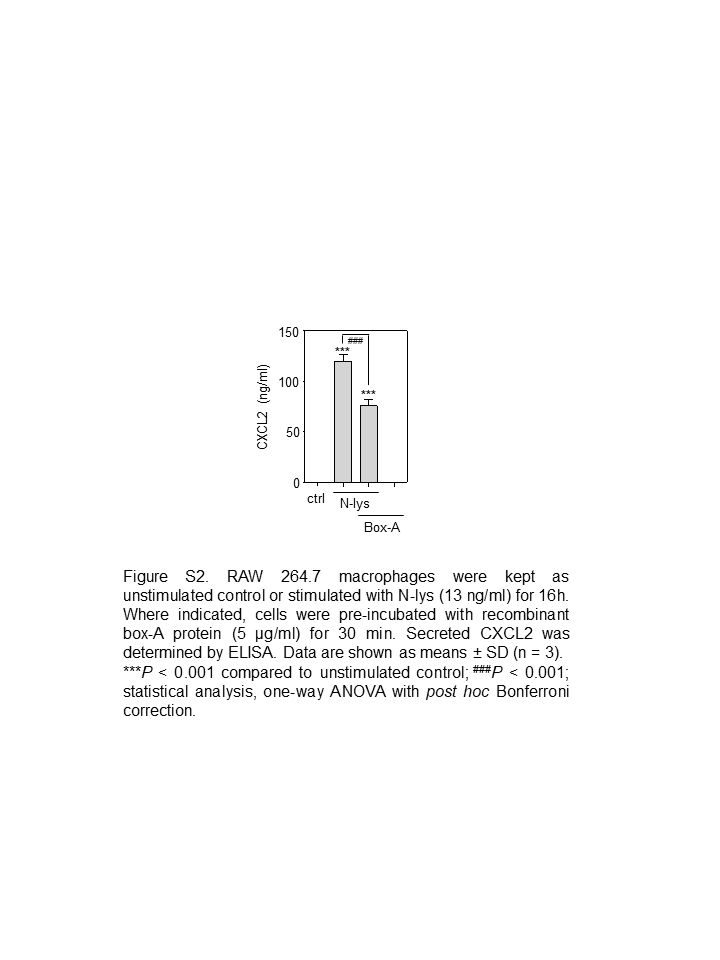

Supplement: Supplementary file 2 — Fig S2 [file JCMM-24-10356-s002.tif]

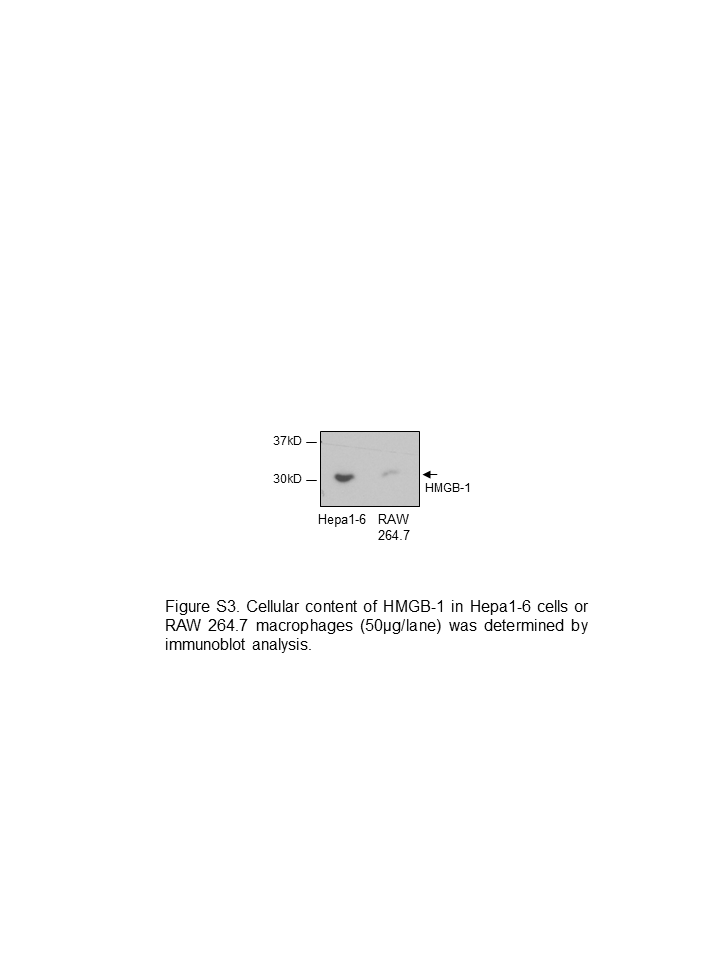

Supplement: Supplementary file 3 — Fig S3 [file JCMM-24-10356-s003.tif]
